# Uncovering the multifaceted roles played by neutrophils in allogeneic hematopoietic stem cell transplantation

**DOI:** 10.1038/s41423-020-00581-9

**Published:** 2020-11-17

**Authors:** Cristina Tecchio, Marco Antonio Cassatella

**Affiliations:** 1 Department of Medicine, Section of Hematology and Bone Marrow Transplant Unit, University of Verona, Verona, Italy; 2 Department of Medicine, Section of General Pathology, University of Verona, Verona, Italy

**Keywords:** neutrophils, allogeneic hematopoietic stem cell transplantation (alloHSCT), alloHSCT-associated complications, low-density neutrophils (LDNs), normal-density neutrophils (NDNs), Neutrophils, Allotransplantation

## Abstract

Allogeneic hematopoietic stem cell transplantation (alloHSCT) is a life-saving procedure used for the treatment of selected hematological malignancies, inborn errors of metabolism, and bone marrow failures. The role of neutrophils in alloHSCT has been traditionally evaluated only in the context of their ability to act as a first line of defense against infection. However, recent evidence has highlighted neutrophils as key effectors of innate and adaptive immune responses through a wide array of newly discovered functions. Accordingly, neutrophils are emerging as highly versatile cells that are able to acquire different, often opposite, functional capacities depending on the microenvironment and their differentiation status. Herein, we review the current knowledge on the multiple functions that neutrophils exhibit through the different stages of alloHSCT, from the hematopoietic stem cell (HSC) mobilization in the donor to the immunological reconstitution that occurs in the recipient following HSC infusion. We also discuss the influence exerted on neutrophils by the immunosuppressive drugs delivered in the course of alloHSCT as part of graft-versus-host disease (GVHD) prophylaxis. Finally, the potential involvement of neutrophils in alloHSCT-related complications, such as transplant-associated thrombotic microangiopathy (TA-TMA), acute and chronic GVHD, and cytomegalovirus (CMV) reactivation, is also discussed. Based on the data reviewed herein, the role played by neutrophils in alloHSCT is far greater than a simple antimicrobial role. However, much remains to be investigated in terms of the potential functions that neutrophils might exert during a highly complex procedure such as alloHSCT.

## Introduction

Allogeneic hematopoietic stem cell transplantation (alloHSCT) has become a well-established life-saving procedure for selected patients with hematological malignancies, inborn errors of metabolism, or bone marrow (BM) failure syndromes.^1^ Briefly, alloHSCT consists of transferring hematopoietic stem cells (HSCs) from either a human leukocyte antigen (HLA)-identical sibling, an HLA-matched unrelated donor, or even an alternative donor [haploidentical-related, mismatched-unrelated, cord blood (CB)] to a patient who has been prepared with a conditioning regimen.^2^ Infusion of HSCs is followed by a pre-engraftment phase, which is characterized by a pancytopenia that lasts ~10–20 days, depending on the HSC source, donor type, conditioning regimen, eventual granulocyte-colony-stimulating factor (G-CSF)-administration, graft-versus-host disease (GVHD)-prophylaxis regimen, or concurrent infectious/noninfectious complications.^3,4^ Engraftment, a crucial phase of alloHSCT, is traditionally defined as the first of three consecutive days during which the patient shows an absolute neutrophil count higher than 0.5 × 10^9^/l, along with sustained platelet (>20 × 10^9^/l) and hemoglobin levels (>80 g/l), and does not require transfusion.^4^ After the engraftment, the donor-derived immune system undergoes a reconstitution phase consisting of a progressive acquisition of competences that relies on the recovery of innate immunity cells during the first weeks, and of adaptive immune cells in the subsequent months.^3,5–7^ Neutrophils are therefore among the first cells to reconstitute, accounting for almost all peripheral blood cells in the first weeks after alloHSCT.^3^ The other cells present during this period are monocytes, natural killer (NK) cells, dendritic cells (DCs), and residual T cells infused with the graft product and undergoing peripheral expansion^3,7^. In patients affected by hematological malignancies, alloHSCT effectiveness also relies on tumor eradication, which depends on both the conditioning regimen and, mostly, graft-versus-tumor (GVT) effects mediated by donor-derived T cells.^2,8,9^ Notably, alloHSCT is characterized by several variables, including (1) the intensity of the conditioning regimen (i.e., myeloablative versus reduced-intensity versus nonmyeloablative); (2) the HSC source [i.e., BM versus mobilized peripheral blood versus CB]; and (3) the type of immunosuppressive regimen [i.e., cyclosporine (CSA) or tacrolimus with a short methotrexate course, CSA and mycophenolate mofetil, eventual anti-thymocyte globulins (ATG), and post-alloHSCT cyclophosphamide].^10^

**Fig. 1:**
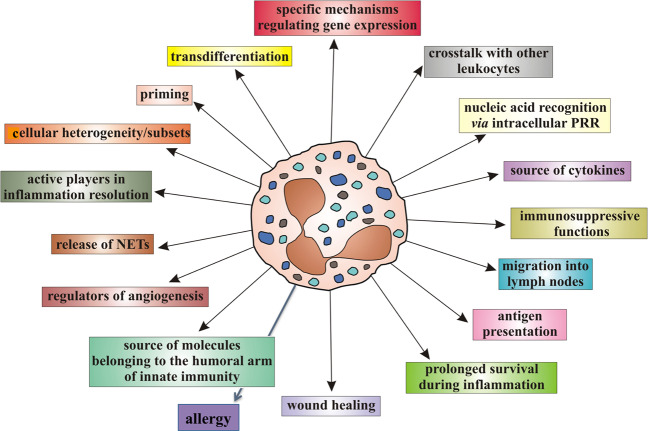
Biologic activities of neutrophils that have been discovered in recent decades

**Fig. 2:**
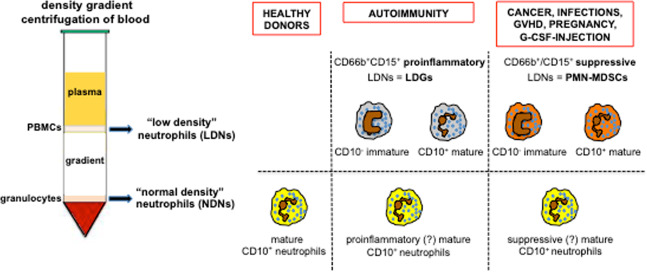
Heterogeneity of neutrophils. After centrifugation of blood from healthy donors over a density gradient, granulocytes typically sediment on top of the erythrocyte layer, while mononuclear cells (i.e., PBMCs) sediment at the interface between the density gradient layer and the plasma. Granulocytes include a homogeneous population of resting mature CD10^+^ neutrophils (conventionally called “normal-density” neutrophils, NDNs) and a variable percentage of eosinophils (not shown).^18^ However, density gradient centrifugation of blood from both patients with diseases and healthy donors may lead to the detection of heterogeneous populations of “low-density” neutrophils (LDNs) within PBMCs under selected physiological conditions or in response to G-CSF stimulation. LDNs may include activated mature CD10^+^ neutrophils and immature CD10^−^ neutrophils (mostly band cells and metamyelocytes, although a few myelocytes and promyelocytes may be present) at different ratios.^18,29,30^ LDNs may manifest either immunosuppressive or proinflammatory properties.^18,20^ Immunosuppressive CD66b^+^/CD15^+^ LDNs, also known as polymorphonuclear myeloid-derived suppressor cells (PMN-MDSCs), are found in patients with, for instance, cancer,^29,31–36^ infections,^25,37^ or graft-versus-host disease (GVHD),^43,44^ as well as in pregnant women^38,39^ and in G-CSF-treated donors.^30,40–42^ Proinflammatory CD66b^+^/CD15^+^ LDNs, also known as low-density granulocytes (LDGs), are instead found in patients with autoimmune diseases, such as systemic lupus erythematosus.^22^ Similarly, in some inflammatory/pathological settings, CD10^+^ NDNs present in the granulocyte fraction may also display immunosuppressive^18^ rather than proinflammatory properties.^18^ To date, no specific markers have been identified in the various neutrophil populations, which basically express the same phenotype present in NDNs (i.e., HLA-DR^-^/CD11b^+^/CD14^−^/CD15^+^/CD33^dim^)

Neutrophils are no longer viewed as involved only in primary defense against infections.^11^ Recent discoveries of their multifaceted activities (mostly shown in Fig. 1) have in fact made it clear that they also function as key effectors not only in the innate and adaptive immune responses but also in many unexpected physiopathological processes, such as angiogenesis,^12^ immune-mediated diseases,^13^ and cancer.^14^ In addition, it has been recently demonstrated that, among the multiple functions exerted, neutrophils are also able to engage in crosstalk with other leukocytes,^15^ to migrate into lymph nodes (LNs)^16^ and even to function as antigen-presenting cells (APCs).^17^ Neutrophils are also emerging as highly versatile cells, being able to acquire different, often opposite, functional capacities, depending on the physiological or pathological context and on their differentiation/activation status.^18^ Accordingly, different heterogeneous populations of mature or immature neutrophils displaying distinct functions have recently been identified, characterized, and named, even though no general consensus exists on whether they unequivocally represent definitive populations.^18–20^ Figure 2 schematizes the current knowledge on the heterogeneity of neutrophils on the basis of their function and intrinsic density. In fact, besides the phenotype, one of the most often used criteria to distinguish neutrophil populations consists of defining their ability to exert either immunosuppressive actions (i.e., inhibition of the proliferation, or the production of IFN-γ, by activated T cells)^21^ or to display enhanced proinflammatory functions [i.e., increased capacity to produce proinflammatory cytokines and/or to release neutrophil extracellular traps (NETs)].^22^ Cell density properties are uncovered by centrifuging blood or other biological samples over commercial gradients (for instance, Ficoll). Accordingly, mature neutrophils from healthy donors (HDs) are often named “normal-density neutrophils” (NDNs) and represent the cells that sediment as a white cell layer over the erythrocytes (Fig. 2). Patient NDNs may become activated in vivo and in turn acquire immunomodulatory functions that may be either immunosuppressive^23–25^ or proinflammatory/immunoactivating,^26^ depending on the disease.^22,27,28^ Populations of circulating neutrophils from patients may display a lower density than NDNs as a result of intrinsic or external factors (such as their immaturity or their in vivo activation, respectively), which may lead them to localize within peripheral blood mononuclear cells (PBMCs) after density gradient centrifugation. These neutrophils are consequently named low-density neutrophils (LDNs) (Fig. 2) and usually consist of a mixture of mature and immature neutrophils.^18,29,30^ LDNs may also display distinct immunomodulatory properties, depending on the disease or other context. For instance, LDNs are highly proinflammatory in patients with autoimmune diseases, in which they are also known as low-density granulocytes (LDGs) (Fig. 2).^22^ By contrast, in patients with cancer^29,31–36^ or infections,^25,37^ in women during pregnancy,^38,39^ and in HDs administered with G-CSF^30,40–42^ or in alloHSCT (as described below),^43,44^ LDNs are immunosuppressive and are conventionally called polymorphonuclear myeloid-derived suppressive cells (PMN-MDSCs) or granulocytic MDSCs (G-MDSCs).

In virtue of accumulating evidence of the plasticity of neutrophils and their ability to influence adaptive immunity,^11,18^ it is quite surprising that neutrophils have been largely overlooked in the context of alloHSCT. Herein, we review the most relevant studies centered on the features, functions and roles played by human neutrophils in alloHSCT, in both the donor [during peripheral blood hematopoietic stem cell (PBSC) mobilization] and the recipient (throughout the different phases of alloHSCT). The contribution of neutrophils to alloHSCT-related complications will also be reviewed. Studies from mouse models involving neutrophils and supporting and/or reproducing alloHSCT-associated clinical or pathological conditions will also be mentioned. While the issue of neutrophil features and functions in alloHSCT is of utmost interest, some caveats and problems should be taken into account. For instance, with regard to in vivo experiments, attention should be paid to the fact that preclinical models are always only partially representative of the complexity of the alloHSCT procedure, especially in terms of conditioning, immunosuppressive prophylactic treatment, and concomitant drug administration.^45^ On the other hand, neutrophils isolated from human peripheral blood or mouse BM for in vitro studies are, according to the literature, not always purified at very high levels (<97%); they are therefore often contaminated by mononuclear cells, which may produce confounding results, especially in terms of gene expression and cytokine release.^46^ Similarly, neutrophils must be carefully and rapidly processed in lipopolysaccharide (LPS)-free medium and reagents to avoid results from nonspecifically activated cells in in vitro experiments. Finally, with respect to ex vivo studies, it is worth noting that alloHSCT patient cohorts are usually not very homogenous, as they may differ in terms of intensity of conditioning, HSC source, donor type, concomitant comorbidities, and especially immunosuppressive treatment. As a consequence, a reliable generalization of the results observed in ex vivo studies, which include heterogeneous patient populations, could be questionable in some ways. Given these limits, the reader will notice that the literature on alloHSCT, unlike that from other clinical settings, has only partially covered the biology of neutrophils and leaves many related questions unanswered. In any case, the information herein revised allows us to conclude that, in alloHSCT, neutrophil functions far exceed those of simple phagocytes involved solely in antimicrobial activities.

## Functions and heterogeneity of neutrophils during, and after, G-CSF-induced PBSC mobilization in healthy donors

PBSCs currently represent the most commonly used HSC source for adult alloHSCT.^47^ These cells are collected by apheresis from the peripheral blood of HDs who have been previously administered recombinant G-CSF for 4–5 days (hereafter, GDs).^48^ The term “mobilization” refers to the procedure that favors a G-CSF-induced forced egression of HSCs from the BM into peripheral blood as PBSCs before their harvest by apheresis into apheretic graft products.^48^ Obviously, even though administered for HSC-mobilizing purposes, G-CSF necessarily also impacts the activation status and functions of neutrophils and their precursors. As described below, two aspects of GD neutrophils have been the focus of investigation by researchers: (1) the role of BM neutrophils as mediators of PBSC mobilization during G-CSF administration and (2) the heterogeneity and functions of peripheral blood neutrophils at the end of the G-CSF-mobilizing treatment period and in the apheresis product.

### Role of BM neutrophils as mediators of PBSC mobilization during G-CSF administration

**Fig. 3:**
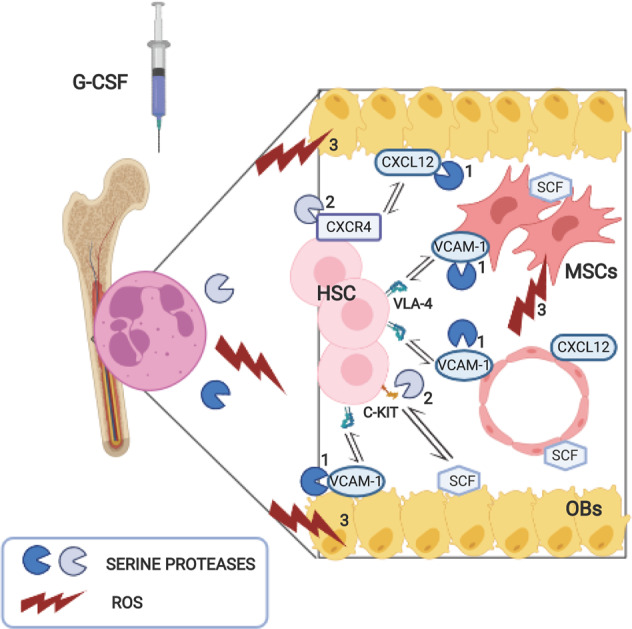
Graphic representation of the proposed mechanisms whereby neutrophils mediate peripheral blood hematopoietic stem cell (PBSC) mobilization under G-CSF treatment. The depicted mechanisms are as follows: 1 release of serine proteases, which in turn cleave and inactivate adhesion molecules (i.e., VCAM-1) and chemokines (i.e., CXCL12) expressed by stromal cells, retaining HSCs in the BM niche;^52,54,56^ 2 release of proteases that cleave CXCR4 (the CXCL12 chemokine receptor) and C-KIT (the SCF receptor) on HSCs;^55,57^ and 3 production of ROS, which in turn induce apoptosis of MSCs and OBs and lead to the loss of CXCL12, SCF, and VCAM-1, which are crucial for HSC retention in the BM niche.^58^ HSCs hematopoietic stem cells, MSCs mesenchymal stromal cells, OBs osteoblasts, VCAM-1 vascular cell adhesion molecule-1, CXCL12 C-X-C motif chemokine ligand 12, CXCR4 C-X-C chemokine receptor 4, C-KIT tyrosine kinase receptor C-KIT, ROS reactive oxygen species, SCF stem cell factor

As extensively reviewed by Tay et al.^49^ conflicting data are present in the literature concerning the participation of neutrophils in G-CSF-induced PBSC mobilization. In fact, it has been shown that G-CSF does not directly act on HSCs (except for inducing their expansion in BM, though with reduced self-renewal capacity^50^ and survival under stressful conditions^51^) but rather functions indirectly through other BM-resident cells expressing G-CSF receptors.^52,53^ Accordingly, based on studies regarding PBSC mobilization in HDs and mouse models, neutrophils were initially indicated as hematopoietic intermediates of G-CSF. In this context, neutrophil-derived serine proteases were found to cleave and inactivate adhesion molecules and chemokines [respectively, vascular cell adhesion molecule-1 (VCAM-1)^52,54^ and C-X-C motif chemokine ligand 12 (CXCL12)^55^] that, under steady state, retain HSCs in the BM niche (Fig. 3, mechanism 1). Consistently, BM specimens from G-CSF-mobilized mice were characterized by a sharp increase in mature neutrophils and their precursors^54^ and a loss of VCAM-1 expression in stromal cells indicated by IHC analysis.^52,54^ In vitro, extracellular BM fluids from the same mice were found to cleave VCAM-1 similarly to supernatants from human peripheral blood neutrophils, further indicating neutrophil elastase (NE) and cathepsin G (CG) as the main serine proteases involved in G-CSF-induced HSC mobilization.^52,54^ In keeping with the aforementioned data, serum levels of soluble VCAM-1 and NE were increased in G-CSF-mobilized patients.^52^ G-CSF-induced HSC mobilization was also found to be associated with neutrophil-mediated cleavage of the N-terminus of CXCR4 (namely, the receptor for CXCL12) in both human and murine HSCs, either resident in BM or mobilized in peripheral blood^55^ (Fig. 3, mechanism 2). The concentration of CXCL12 in the BM of mobilized HDs and mice was reported to decrease concomitant with the accumulation of neutrophil-derived serine proteases.^55,56^ The latter enzymes were also reported to cleave the tyrosine kinase C-KIT, namely, the stem cell factor (SCF) receptor, in murine HSCs^57^ (Fig. 3, mechanism 2). Moreover, murine neutrophils, expanded in BM upon G-CSF administration, were found to induce apoptosis of mesenchymal stromal cells (MSCs) and osteoblasts via the production of reactive oxygen species (ROS)^58^ (Fig. 3, mechanism 3). As a consequence, the expression of CXCL12, SCF, and VCAM-1, all molecules essential for HSC retention in the BM endosteal niche, was found to drastically decrease.^58^ Overall, the studies reported above point to a crucial role of G-CSF-stimulated neutrophils (and their precursors) in favoring the egress of HSCs from the BM niche through the release of serine proteases or ROS. However, the putative central role of neutrophils in G-CSF-induced PBSC mobilization has been questioned by other studies. In fact, mice partially lacking the expression of NE, CG, or dipeptidyl peptidase-I were shown to mobilize HSCs in response to G-CSF to the same degree as wild-type mice.^59^ More recent evidence seems to restrain the role of neutrophils in HSC mobilization, in turn indicating that their protease release is only one of the multiple events that follow the G-CSF-induced shutdown of the endosteal niche.^60^ Although G-CSF administration currently represents the standard approved regimen for PBSC mobilization in HDs,^61^ research to identify alternative mobilizing agents is ongoing. For instance, although still under investigational use in HDs, AMD3100/plerixafor (which acts by inhibiting CXCR4 expressed by HSCs), administered either alone^62,63^ or in combination with G-CSF,^64^ represents an effective, direct HSC-mobilizing agent that does not require neutrophil involvement. Moreover, based on data from humans and murine models, plerixafor controls the trafficking of neutrophils by inducing their demargination from the lungs and blocking their homing to the BM^65^ or, alternatively, by favoring their egress from the BM.^66^ Finally, combined mobilization strategies targeting either very late antigen-4 (VLA4, an adhesion molecule that contributes to the retention of HSCs in the BM niche) or CXCR2 (expressed by stromal cells and neutrophils) have been explored at the preclinical level in mouse models.^67^ In this context, neutrophils have been hypothesized to boost the VLA4 agonist-induced mobilization of HSCs through the release of proteases.^67^ In addition, upon stimulation with CXCR2 agonists, neutrophils could also favor the egress of HSCs from the BM niche through interplay with endothelial cells.^67^

### Heterogeneity and functions of peripheral blood neutrophils at the end of the G-CSF-mobilizing treatment and in the apheresis product

**Fig. 4:**
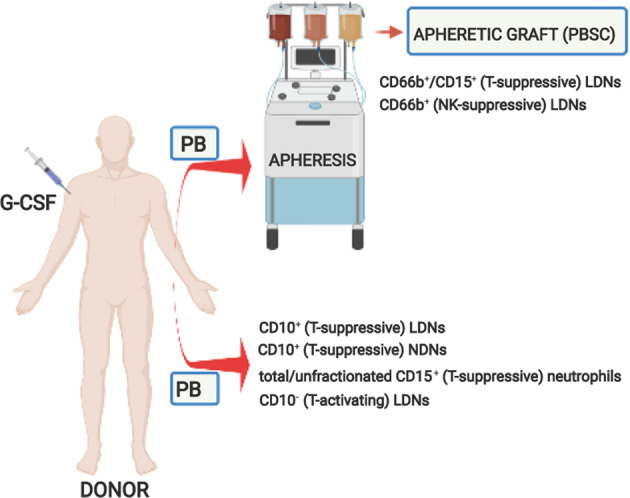
Graphic overview of the neutrophil populations isolated from the peripheral blood of G-CSF-treated healthy donors, as well as from their apheretic graft products (PBSCs). Data are extrapolated from the literature. PB peripheral blood, PBSCs peripheral blood stem cells. Neutrophil populations are CD66b^+^/CD15^+^ (T-suppressive) LDNs^40^ (immunophenotype inferred from morphological data); CD66b^+^ (NK-suppressive) LDNs;^42^ CD10^+^ (T-suppressive) LDNs;^30^ CD10^+^ (T-suppressive) NDNs;^30^ total/unfractionated CD15^+^ (T-suppressive) neutrophils;^41^ and CD10^−^(T-activating) LDNs^30^

Ex vivo studies analyzing the phenotype and functions of peripheral neutrophils following G-CSF administration for mobilization purposes are relatively rare. This is due not only to the prevalent interest of both researchers and clinicians in the yield and quality of HSCs to be transplanted but also to a long-standing view of neutrophils as short-lived cells, simply contaminants of the apheresis graft products. Nonetheless, important data have recently emerged on the immunomodulatory properties acquired by peripheral human or mouse neutrophils following G-CSF-induced PBSC mobilization. In fact, neutrophils from GDs have been found to display a number of features distinguishing them from those of HDs, other than appearing as heterogeneous populations of both immature and mature activated cells with distinctive characteristics^68^ (Fig. 4).

As already mentioned, studies carried out in cancer patients originally showed that LDNs/PMN-MDSCs correlate in vivo with reduced T-cell receptor ζ chain expression and ex vivo with decreased production of cytokines such as interferon-γ (IFN-γ), interleukin-4 (IL-4), tumor necrosis factor-α (TNF-α), and IL-2 by phorbol-myristate acetate (PMA)- and ionomycin-stimulated T cells.^28^ Interestingly, neutrophils with similar properties were also described by Vasconcelos et al.^40^ in the peripheral blood of GDs (Fig. 4). Accordingly, T-cell function, determined by IFN-γ and IL-4 production upon 4 h of treatment with PMA or ionomycin, was significantly reduced in apheresis products from GDs compared to BM and peripheral blood from HDs.^40^ Furthermore, a detailed morphological analysis revealed that the mononuclear fraction of the apheresis products was mainly composed of neutrophils (i.e., LDNs/PMN-MDSCs), whose death after 48 h of in vitro culture was accompanied by a restoration of IFN-γ production by T cells.^40^ Later, Luyckx et al.^41^ identified in unfractionated peripheral blood sampled from GDs (Fig. 4) a population of mature SSC^hi^CD33^int^CD14^low^CD15^+^ neutrophils able to negatively regulate the alloreactivity of T cells in vitro. Marini et al.^30^ further investigated the characteristics of immunosuppressive neutrophils in terms of phenotypes and functions in a study comparing peripheral blood NDNs and LDNs from GDs versus NDNs from HDs. Based on CD10 expression as a marker of mature neutrophils, both NDNs and LDNs were found to contain a heterogeneous population composed of CD66b^+^CD10^−^ immature neutrophils and CD66b^+^CD10^+^ mature neutrophils^30^ (Fig. 4). Importantly, while CD66b^+^CD10^−^ LDNs were found to display immunostimulating functions, CD66b^+^CD10^+^ neutrophils resulted in immunosuppressive functions^30^ (Fig. 4). Moreover, CD66b^+^CD10^+^ neutrophils isolated by sorting from LDNs, NDNs, or whole blood of GDs were found to inhibit CD3/CD28-induced proliferation of and IFN-γ production by T cells in vitro via CD18-mediated contact-dependent arginase 1 (Arg-1) release.^30^ As expected, CD66b^+^CD10^+^ neutrophils isolated from NDNs of HDs were not effective in modulating any T-cell function.^30^ In addition, CD66b^+^CD10^+^ cells from GDs displayed an activated phenotype that was characterized by lower CD16 and higher CD54/ICAM-1, CD11c, and CD35 expression than was observed in CD66b^+^CD10^+^ neutrophils from unstimulated HDs.^30^ Although CD10 itself is known to be an activation marker,^69,70^ the lack of T-cell suppressive activities by CD10^+^ neutrophils from HDs suggests that in vivo G-CSF induces complex cell reprogramming rather than a general activation status. Interestingly, NDNs from HDs were found not to acquire any immunosuppressive functions upon treatment with G-CSF in in vitro experiments.^30^ Taken together, the data provided by Marini et al.^30^ indicate that the immunosuppressive properties of CD66^+^CD10^+^ mature neutrophils from GDs are strictly dependent on in vivo exposure to G-CSF and eventually on other G-CSF-induced factors, such as IL-6, IL-10, granulocyte-macrophage colony-stimulating factor (GM-CSF), transforming growth factor-β (TGF-β), and prostaglandin-E_2_ (PGE_2_), which are known to induce PMN-MDSC differentiation.^19^ However, according to the same authors, the immunosuppressive properties of CD66^+^CD10^+^ mature neutrophils from GDs are not necessarily associated with their sedimentation into the low-density fraction of the gradient.^30^ More recently, Tumino et al.^42^ have identified in αβT-cell- and B cell-depleted apheresis products from G-CSF mobilized haploidentical donors (Fig. 4) a highly enriched population of PMN-MDSCs (i.e., low-density CD45^+^Lin^−^HLA-DR^−/low^CD33^+^CD11b^+^CD14^−^CD66b^+^ cells) able to exert sharp inhibition of the cytotoxic activity of autologous NK cells against recipient leukemic blasts in vitro. Based on these findings, the authors hypothesized that the PMN-MDSCs in αβT-cell- and B cell-depleted apheresis products might inhibit the graft-versus-leukemia activity exerted in the recipient by the coinfused mature NK cells.^42^ Interestingly, the immunosuppressive activity of the same PMN-MDSCs was found to involve the production of indoleamine 2,3-dioxygenase (IDO), PGE_2_, and exosomes.^42^

Overall, although analyzing different sample types, i.e., apheresis products^40,42^ versus peripheral blood,^30,41^ and employing different characterization methods and in vitro analyses, all studies described above point to the ability of G-CSF to induce heterogeneous populations of immature and mature neutrophils endowed with immunomodulating properties in vivo (Fig. 4). To what extent these cells copurify with HSCs during the apheretic procedures (and how much the apheresis instrumentation settings may condition this copurification), how long-lived they are after infusion, and how they functionally behave after alloHSCT, under an altered immunological context and concomitant with the administration of immunosuppressive drugs, are all currently unknown. With regard to the first issue, Lv et al.^71^ evaluated the composition (in terms of MDSC subtypes) of BM and peripheral blood from 20 HDs before and after the administration of G-CSF for mobilization, as well as the composition of the corresponding apheretic grafts. According to these authors, SSC^low^CD11b^+^CD33^+^CD14^+^HLA-DR^−^Lin^−^CD15^dim^CD16^−^ monocytic (M)-, SSC^high^CD33^+^CD11b^dim^CD16^−^Lin^−^ promyelocytic (P)-, and SSC^high^CD11b^+^CD33^dim^CD15^+^HLA-DR^−^Lin^−^ granulocytic (G)- MDSCs were significantly expanded in donor BM and peripheral blood after G-CSF treatment.^71^ However, the apheretic grafts were found to contain lower percentages of G- and P-MDSCs than mobilized peripheral blood samples.^71^ By contrast, M-MDSCs were enriched in the apheretic grafts, indicating that the apheretic process was able to preferentially retain M-MDSCs compared to P- and G-MDSCs.^71^ In partial contrast with these data, αβT-cell- and B cell-depleted apheresis products from haploidentical donors were found to contain significantly higher percentages of PMN-MDSCs (defined as low-density CD45^+^Lin^-^HLA-DR^−/low^CD33^+^CD11b^+^CD14^−^CD66b^+^ cells) than M-MDSCs.^42^

Other issues that need to be clarified are whether the presence of immunosuppressive neutrophils in G-CSF-mobilized graft products may affect outcomes in alloHSCT patients, exerting either a preventive effect against acute GVHD (aGVHD) or an unwanted inhibition of GVT. In their evaluation of 62 patients undergoing haploidentical HSCT from G-CSF-primed BM plus G-CSF-mobilized PBSCs, Lv et al.^71^ performed a multivariate analysis that showed an inverse correlation between the absolute number of P-MDSCs or M-MDSCs, but not G-MDSCs, infused and the occurrence of grade II–IV aGVHD and extensive chronic GVHD (cGVHD).

Mature, low-density, immunosuppressive neutrophils with protective effects against aGVHD have also been described in mouse models. Accordingly, initial data showed that the low-density fraction obtained by density gradient centrifugation of splenocytes from G-CSF-treated mice contains a population of mostly mature (Gr1^+^SCA1^−^) neutrophils, accounting for 20% of the whole low-density cell fraction (i.e., LDNs).^72^ LDNs able to inhibit IFN-γ production by T cells via a mechanism dependent on hydrogen peroxide were also generated in vitro by treatment of mouse neutrophils with G-CSF.^72^ Importantly, in a model of experimental aGVHD obtained by injecting splenocytes as an allogeneic T-cell source, the Gr1^+^ cells present in splenocytes from G-CSF-treated mice were protective toward aGVHD.^72^ In fact, the injection of splenocytes depleted of Gr1^+^ cells was associated with the development of aGVHD.^72^ More recently, Perobelli et al.^73^ extended these results by demonstrating that the protection against aGVHD by splenocytes from G-CSF-treated mice relies on the presence of suppressive IL-10^+^ LDNs able to induce post transplant expansion of T regulatory (CD4^+^CD25^+^Foxp3^+^) cells (Tregs)^73^. Interestingly, IL-10^+^ LDNs were characterized by the authors as activated Ly6C^−^Ly6G^+^ cells with elevated phagocytic capacity, elevated peroxide production, low myeloperoxidase (MPO) activity, low cytoplasmic granule content, low expression of MHC class II and costimulatory molecules, and low Arg-1 expression.^73^ Since the features of these IL-10^+^ LDNs do not strictly fit the available description of any known human neutrophil population, the authors referred to these IL-10^+^ LDNs simply as activated and suppressive “G-neutrophils”.^73^ For a detailed review of all MDSC types in alloHSCT, either in humans or in mice, the reader is invited to read the article by D’Aveni et al.^74^

Taken together, these findings point to complex immunomodulatory roles acquired by neutrophils following G-CSF administration to HDs for mobilization. However, further studies are urgently required to investigate, prospectively and in more homogeneous cohorts of donors and patients, the presence of immunomodulating neutrophils in graft products, the extent of their persistence in recipients after PBSC infusions, and their relationship with aGVHD occurrence and GVT effects.

## Neutrophil immunophenotype and properties during immune reconstitution in alloHSCT recipients

As already mentioned, the HSC engraftment phase of alloHSCT is followed by the reconstitution of donor-derived innate and adaptive immune cells. Neutrophils are among the first cells to reconstitute and thus represent the large majority of circulating cells, and almost the sole cells of the immune system on which the patient can rely, in the first weeks after alloHSCT.^3,5^ However, the functions and/or features of neutrophil reconstitution after alloHSCT have been rarely investigated.^75^ In addition to the old, classic view of neutrophils as simple phagocytic cells unable to perform additional biological functions, several impeding factors (including patient heterogeneity in terms of concomitant clinical complications and therapies) have hampered the implementation of studies aimed at defining neutrophil phenotypes and properties throughout immune reconstitution. Accordingly, peripheral blood neutrophils of alloHSCT recipients have been traditionally evaluated only in terms of their numbers by routine blood count analysis to assess the achievement of engraftment and the rescue of innate immunity against infections. Concerning their immunophenotypes, the little data available in the literature indicate that the large majority of reconstituting neutrophils consist of terminally differentiated CD10^+^ and CD16^+^ cells, expressing CD11b levels similar to those of HD neutrophils.^76,77^ Only a few reports have specifically addressed the issue of immune reconstitution in light of the most recent advances in neutrophils as heterogeneous cell populations.^18,20^ In a study evaluating G- and M-MDSC subsets in the peripheral blood of 26 patients before conditioning and at six time points during the first 3 months after alloHSCT, Guan et al.^78^ identified a population of CD33^+^CD15^+^CD66b^+^ G-MDSCs that, by days +27 to 29 post-alloHSCT, reached absolute number levels in the same range of those found in patients during the preconditioning period. Interestingly, these cells were functional, as they were found to suppress CD4^+^IFN-γ^+^Th1 cells, as well as to promote the development of CD4^+^CD25^+^Foxp3^+^ Treg cells in coculture experiments with CD3/CD28-stimulated third-party CD4^+^ T cells.^78^ Consistent with these data, the G-MDSC number at preconditioning and the ratio of G-MDSCs at days 27–29 to the number at preconditioning were found to inversely correlate with grades II–IV aGVHD.^78^

In a preliminary prospective study analyzing peripheral blood from 8 HDs and 39 patients at days +21, +42, +60, +90, and +180 after alloHSCT, we found an increased frequency of LDNs in the peripheral blood of alloHSCT patients compared to HDs and a progressive reduction of the immature CD66b^+^CD10^−^ fraction of LDNs in patients without GVHD.^44^ Importantly, consistent with a T-cell-activating phenotype of immature neutrophils,^30^ the frequency of CD66b^+^CD10^−^ LDNs was significantly higher in patients affected by aGVHD than in patients without this complication.^44^ Based on the previous two studies,^44,78^ it seems reasonable to hypothesize that HSC infusion is followed by the reconstitution of different populations of neutrophils endowed with immunomodulating properties. Obviously, further research is needed to establish the real participation of such neutrophil populations in the pathophysiology of GVHD and/or GVT, as well as to elucidate their role as potential therapeutic targets.

Contrasting data concerning the maintenance of functional mature neutrophils during immune reconstitution have been published.^75^ In fact, while some authors have reported that the functions of neutrophils are unaffected, others have described that they are reduced in the first 1–12 months after alloHSCT, especially in concomitance with GVHD or other alloHSCT-related complications. For example, neutrophil chemotaxis has been reported as either reduced^79–81^ or unaffected^82^ relative to that of cells from HDs. Additionally, phagocytosis has been described to be either decreased^83^ or normal.^77,81,84,85^ Impaired respiratory burst activity in response to discrete stimuli has been reported to occur in the first months after alloHSCT by some authors,^82,85,86^ but not by others.^77,87^ More recent studies have also pointed to a reduced capacity of neutrophils from alloHSCT patients to release NETs in response to PMA^88,89^ or LPS.^76^ NETs are extracellular meshes composed of neutrophil-derived chromosomal DNA, histones, and granule proteins that provide a scaffold for high local concentrations of antimicrobial components to kill microbes extracellularly.^90^ NET deployment is coupled to plasma membrane disruption and culminates in a form of cell death called “NETosis”.^91^ As with other neutrophil-derived defensive products, NETs may also be highly toxic to the host, and their release may contribute to cellular injury and organ dysfunction.^92^ Once again, the abovementioned data were obtained from heterogeneous cohorts of patients in terms of GVHD prophylaxis regimens, which might explain the conflicting results.

The impact of immunosuppressive drugs on neutrophil functions has been highlighted only recently. Although pharmacological agents, such as CSA and tacrolimus/FK-506 (calcineurin inhibitors, CNIs) and rapamycin [the mammalian target of rapamycin (mTOR) inhibitor], have been included in aGVHD prophylaxis schemes with the primary aim of inhibiting T-cell alloreactivity,^10^ accumulating evidence points to their interference with the functionality of innate immune cells. This is the case for CNIs—part of standard aGVHD prophylaxis regimens—which inhibit the calcineurin-mediated activation of the transcription factor known as calcineurin/nuclear factor of activated T cells (NFAT), as well as the transcription and production of many T-cell-derived cytokines, including IL-2.^93^ In vitro evidence has also indicated that the calcineurin–NFAT pathway activates and controls important anti-pathogen functions in human and murine myeloid cells, which, in turn, might be impaired by CNIs.^94,95^ According to initial studies, in fact, murine neutrophils treated with CSA in vitro were found to be deficient in *Candida albicans* killing activity, similar to neutrophils carrying a conditional deletion of the calcineurin B gene, thus indicating some involvement of the calcineurin–NFAT signaling pathway in anti-fungal actions.^94^ Notably, the CSA-mediated impairment of fungal killing was found not to be associated with reductions in phagocytosis, the production of ROS, the production of nitric oxide (NO) or MPO degranulation but rather to an as-yet unidentified impaired killing mechanism.^94^ By contrast, the triggering of the calcineurin/NFAT pathway by zymosan (a yeast component) was found to activate NFAT-dependent genes such as IL-10, Cox2, Egr1, and Egr2 in murine neutrophils.^94^ CSA was also found to downmodulate, in murine neutrophils, the NFAT-dependent transcription of the nucleotide-binding oligomerization domain 1 (nod1) gene, which encodes an intracellular pattern recognition receptor involved in neutrophil sensing of gram-negative bacteria and phagocytic response.^95^ CSA and the tacrolimus analog ascomycin were subsequently found to dose-dependently impair the capacity of human neutrophils to release NETs in vitro in response to CXCL8 or ionomycin^96^ but not in response to LPS.^97^ Very importantly, an ex vivo study comparing the anti-*Aspergillus fumigatus* (AF) actions exerted by neutrophils from alloHSCT patients (at engraftment and 2, 6, and 10 months after HSC infusion) and from their respective HDs showed a reduced activity toward AF hyphal growth by patient neutrophils that persisted until CSA or tacrolimus administration was stopped or greatly reduced.^87^ Notably, in the presence of significant therapeutic CSA or tacrolimus plasma levels, the release of NETs was the only anti-fungal activity impaired.^87^ Taken together, these data indicate that depending on the stimulatory condition, some anti-pathogen functions of neutrophils may become impaired in alloHSCT patients by therapeutic concentrations of CNIs.

Recent studies have established an important role for the mTOR network [composed of mTOR complexes 1 and 2 (mTORC1 and mTORC2)] in controlling and shaping the effector responses of innate immunity cells.^98^ In particular, it has been demonstrated that mTOR is involved in the control of chemotactic migration, constitutive mRNA translation and the expression of proinflammatory cytokines by human and mouse neutrophils.^98^ Although less commonly included in aGVHD prophylaxis schemes than CNIs,^99^ rapamycin has been shown to interfere with crucial neutrophil functions. For instance, in vitro studies have shown that in human neutrophils, rapamycin inhibits actin polymerization and consequently interferes with chemotaxis induced by GM-CSF and, at higher doses, CXCL8^100^ and phosphatidic acid.^101^ In addition, rapamycin can also regulate the release of NETs by human neutrophils in different manners depending on the stimulus.^98^ Accordingly, rapamycin was found to inhibit the effect of LPS on NET release through translational control of HIF-1α, a downstream regulator of the mTOR pathway and a critical modulator of antimicrobial defenses.^97^ By contrast, rapamycin turned out to accelerate the release rate of NETs by neutrophils stimulated in vitro with the bacteria-derived peptide formyl-Met-Leu-Phe (fMLF) via a positive regulation of autophagy.^102^ Interestingly, NET release was not observed in a study analyzing neutrophils obtained from alloHSCT patients under treatment with CNIs (alone or in combination with other immunosuppressive agents) and exposed to rapamycin.^88^ Finally, rapamycin was also shown to impair the IL-23-induced production of IL-22 and IL-17 by murine neutrophils in vitro.^103^ Therefore, according to these in vitro observations, human neutrophils may restrain some anti-pathogen functions when exposed to CNIs or mTOR inhibitors.

Other classes of immunosuppressive agents, including ATG, methotrexate, mycophenolate mofetil, and post-HSCT cyclophosphamide (which may be used in combination with CNIs or rapamycin in aGVHD prophylaxis regimens), might impair neutrophil functions. In addition, the treatment of overt aGVHD may require the use of additional immunosuppressive agents such as steroids,^10^ thus adding further complexity to the evaluation of neutrophil functionality in the first months after alloHSCT. For instance, in a prospective study including 51 patients analyzed at days +30, +90, +180, and +360 after HSCT, Stueheler et al.^86^ observed that neutrophils tended to have a diminished capacity to produce ROS toward *A. fumigatus* in patients with aGVHD under steroid treatment compared to patients without aGVHD. Although ex vivo studies are lacking (with the exception of one by Imbert et al.^87^), it is conceivable to hypothesize that neutrophils of alloHSCT patients become deficient in some of their anti-pathogen functions depending on the pathogen and/or on the immunosuppressant type and plasma levels.

A further complicating factor in the assessment of neutrophil competence during immune reconstitution is presented by G-CSF, which is administered concomitantly to immunosuppressive agents in selected alloHSCT patients, such as those receiving CB^104^ or haploidentical HSCs.^105^ Based on the evidence that the administration of ATG delays CD4^+^ T-cell reconstitution more severely in patients receiving CB plus G-CSF than in those receiving BM only, de Koning et al.^104^ hypothesized that G-CSF might increase the ATG-mediated phagocytic activity of neutrophils toward T cells. Sustaining this hypothesis, neutrophils from GDs were found to display a strikingly higher capacity to phagocytize ATG-targeted T cells in vitro than neutrophils from HDs,^104^ therefore indicating that, in vivo, G-CSF-activated reconstituting neutrophils could be responsible for the delayed T-cell reconstitution observed after CB infusion.

In light of all the aforementioned findings, it seems reasonable that researchers involved in studies that analyze neutrophils and neutrophil populations in the human alloHSCT setting should take into account the multiple confounding effects deriving from the concomitant administration of immunosuppressive agents and/or G-CSF. Therefore, given the higher susceptibility to infections of patients in the first months following alloHSCT, neutrophil functionality throughout immune reconstitution should be more appropriately evaluated in the context of prospective ex vivo studies obtained from homogeneous groups of patients. This would allow a better assessment of the extent of the influence of immunosuppressive agents on neutrophil responses to different stimuli, as well as their dose-dependent effects.

Finally, functional alterations of neutrophils after alloHSCT may also depend on genetic variants of pattern recognition receptors (PPRs) of donor origin.^106,107^ For instance, extensive studies on HDs and their corresponding recipients demonstrated that hyporesponsive Toll-like receptor 4 (TLR4)^106^ or pentraxin 3^107^ genetic variants in HSC donor-derived neutrophils might render alloHSCT patients more susceptible to aspergillosis by affecting anti-fungal neutrophil activity.

## Involvement of neutrophils in alloHSCT-associated complications

**Fig. 5:**
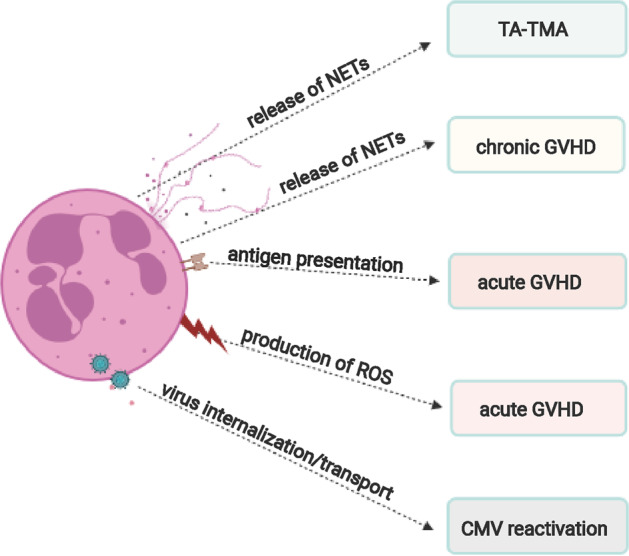
Graphic representation of neutrophil effector functions specifically involved in each of the alloHSCT-associated complications/conditions, according to published data. NETs neutrophil extracellular traps, ROS reactive oxygen species, TA-TMA transplant-associated thrombotic microangiopathy, GVHD graft-versus-host disease, CMV cytomegalovirus. References for TA-TMA (NETs)^110–112^; aGVHD (ROS)^122,125^; aGVHD (antigen presentation)^123^; cGVHD (NETs)^129^; CMV reactivation (virus transport)^140,141^

AlloHSCT is often associated with early and late noninfectious (e.g., vascular endothelial syndromes, aGVHD and cGVHD) and infectious (e.g., CMV reactivation) complications that can threaten patient survival, aside from primary disease relapse. As briefly described below, neutrophils have been described to contribute to the pathogenesis of such complications by virtue of their different functions, including NET release, ROS production, antigen presentation and virus internalization and transport (Fig. 5).

### Vascular endothelial syndromes

Among early noninfectious complications, vascular endothelial syndromes represent a range of early life-threatening complications that often provoke a sudden worsening of patient clinical condition.^108^ Transplant-associated thrombotic microangiopathy (TA-TMA) is one of the most recognized of these entities, together with capillary leak syndrome, engraftment syndrome, and idiopathic pneumonia syndrome.^108^ TA-TMA is caused by a disseminated platelet clumping in microcirculation and is characterized by nonimmune hemolytic anemia, thrombocytopenia, elevated serum lactate dehydrogenase, schistocytosis and severe hypertension and proteinuria.^109^ Consequently, TA-TMA is associated with a poor prognosis and a high mortality rate owing to end-organ damage.^108,109^ The main trigger of TA-TMA is thought to be damage to the endothelium caused by alloHSCT-related procedures (e.g., conditioning regimens, CNIs, or antimicrobial drugs) or their side effects (e.g., infections and aGVHD).^108,109^ Importantly, a NET-mediated involvement of neutrophils in endothelial damage and TA-TMA pathogenesis has been indicated by recent studies,^110–112^ which in turn are based on previous findings obtained in alloHSCT-independent TMA, such as thrombotic thrombocytopenic purpura and hemolytic uremic syndrome.^108,109^ However, prior to listing and discussing the aforementioned studies,^110–112^ some considerations on NETs/NETosis are necessary. In fact, the involvement of NETs/NETosis in disease pathogenesis is currently being investigated in a variety of pathologic conditions. Supportive evidence for NETosis in vivo consists mainly of measuring DNA in blood (or serum/plasma) or tissue. However, NETs are difficult to track, as they must be identified by the detection of three separate molecules that colocalize in their structure (i.e., DNA, histones, and NE attached to the chromatin). Since there is no doubt that cell-free DNA can be found in many disorders, entirely independent of NETs in serum, plasma or tissue, it follows that most of the data described below must be interpreted with caution. Keeping these caveats in mind, Arai et al.^110^ sought to evaluate the predictive significance of NET serum levels with respect to TA-TMA in a cohort of 90 adult patients analyzed before conditioning, at alloHSCT (day 0), and in the fourth week thereafter. These authors quantified serum NETs as double-stranded (ds) DNAs after having observed a significant correlation between dsDNA and MPO-DNA serum levels in a representative group of patients.^110^ Multivariate analysis indicated that dsDNA serum levels were predictive for TA-TMA when either the ratio between day 0 and preconditioning levels or absolute levels at the fourth week were considered.^110^ In addition, immunofluorescence staining of MPO and DNA revealed NET deposits in the glomeruli of kidney specimens from deceased TA-TMA patients.^110^ A subsequent longitudinal study on 103 consecutive alloHSCT pediatric patients (analyzed at days 0, +14, +30, +60, and +100) aimed at exploring the mechanistic link between endothelial injury and TA-TMA demonstrated that DNA levels, measured as a surrogate for NETs, were significantly increased at day +14 in patients who subsequently developed TA-TMA.^111^ Interestingly, neutrophil count and DNA levels were inversely correlated with concomitantly detected serum levels of CXCL8, a chemokine that induces neutrophil chemotaxis and NET release.^111^ Since day +14 neutrophils were found to display a downmodulation of CXCR1 and CXCR2 (interpreted as a result of CXCL8 internalization), the authors hypothesized that CXCL8, released by damaged endothelium, could induce the release of NETs by neutrophils and, in turn, the activation of complement and formation of microthrombi.^111^ In agreement with this hypothesis, children undergoing complement-blocking therapy with eculizumab (a monoclonal antibody targeting C5) for severe TA-TMA displayed significantly lower DNA serum levels at disease resolution/therapy discontinuation than at diagnosis and at 4 weeks into treatment.^111^ Finally, a very recent case–control study analyzing 30 alloHSCT patients with TA-TMA (*n* = 10), aGVHD or cGVHD (*n* = 10), or without any complication (*n* = 10) further clarified that the pathogenesis of TA-TMA relies on crosstalk among NETs, the complement system and the coagulation cascade.^112^ In fact, by simultaneously analyzing markers of NETs (i.e., DNA and MPO-DNA), as well as of complement and coagulation activation, in serum and/or plasma, the authors found that TA-TMA patients had an enhanced release of NETs that was associated with activation of complement and coagulation.^112^ Notably, serum markers of endothelial damage (i.e., soluble thrombomodulin and soluble VCAM-1) were detectable not only in TA-TMA but also in GVHD patients, therefore indicating a possible overlap between the two pathologies.^112^ Overall, although different in terms of design, cohort composition, type of conditioning, GVHD prophylaxis regimen, time-point assessments, and diagnostic criteria, the studies reported above suggest neutrophil involvement in TA-TMA pathogenesis through NET release. This is in agreement with previous in vitro studies showing that human neutrophils exposed to antineutrophil cytoplasmic antibodies (ANCAs) activate the alternative complement pathway via NET release, ultimately damaging the endothelium through the assembly of membrane attack complexes (C5b-9).^113^ NETs have also been reported to activate the coagulation system in both humans and mice. Accordingly, neutrophils from patients affected by ANCA-associated vasculitis, a disease characterized by hypercoagulopathy, were shown to release tissue factor (TF)-expressing NETs.^114^ In a mouse model, thrombus-resident neutrophils were also found to contribute to deep vein thrombosis by activating FXII through NET relase.^115^ Finally, previous in vivo and in vitro findings have shown that both human and mouse neutrophils contribute to the thrombotic process via the release of nucleosomes containing serine proteases involved in the local proteolytic cleavage of tissue factor pathway inhibitor,^116^ the primary inhibitor of the initiation of the coagulation cascade. On the other hand, no data are currently available about the involvement of neutrophils in alloHSCT-related vascular endothelial syndromes other than TA-TMA. For instance, to the best of our knowledge, there is no information available about the involvement of NETs in sinusoidal obstruction syndrome/veno-occlusive disease of the liver, a severe post-alloHSCT syndrome partially related to endothelial damage.^117^ Finally, considering that the release of NETs by neutrophils was reported as impaired in the same post-alloHSCT period (the reader is referred to the section “Neutrophil immunophenotype and properties during immune reconstitution in alloHSCT recipients” in this review), it remains to be clarified whether reconstituting neutrophils may actually release NETs in response to selected disease-associated stimuli.

### Acute GVHD (aGVHD)

GVHD is a major complication of alloHSCT and hence a main cause of post-alloHSCT morbidity and mortality.^118^ It occurs in acute and chronic forms, which can be distinguished according to clinical signs and manifestations that only partially overlap the traditional chronological classification of acute (before day +100) and chronic (after day +100) GVHD.^119^ The acute form of GVHD (i.e., aGVHD) remains a significant cause of morbidity and mortality in alloHSCT recipients.^120^ It substantially consists of severe inflammatory complications resulting from a wide range of immune mechanisms that donor T cells employ to attack recipient tissues recognized as “nonself”.^120^ Indeed, the pathophysiology of aGVHD has been attributed to a three-phase process consisting of (1) initial tissue damage from the conditioning regimen, which in turn leads to (2) activation of host APCs by microbial-associated molecular patterns (MAMPs)^121^ and danger-associated molecular patterns (DAMPs), with activation and proliferation of donor (graft) T cells, resulting in (3) cytotoxic damage to recipient (host) cells and release of inflammatory cytokines.^120^ Interestingly, recent evidence has indicated that neutrophils may have a role in the pathogenesis of human and mouse intestinal aGVHD by activating donor effector T cells, either through the promotion of an inflammatory microenvironment^122^ or by presenting host antigens to the T cells in mesenteric LNs (mLNs).^123^ In fact, in a study using different alloHSCT mouse models, Schwab et al.^122^ demonstrated that, in the conditioning-damaged gastrointestinal tract, the activated neutrophils of the recipient affect the severity of aGVHD via the production of ROS, which in addition to reacting against invading bacteria also damage surrounding/bystander tissue cells. This latter damage was shown to further activate neutrophils to elicit a highly inflammatory environment, in turn promoting T-cell activation and favoring aGVHD development.^122^ Accordingly, antibody-mediated (anti-Ly6G) or genetically determined depletion of neutrophils was found to reduce aGVHD aggressiveness and mortality in the same animal models.^122^ Importantly, the same authors were able to demonstrate that neutrophil recruitment occurs upon translocation of intestinal bacteria into the conditioning-damaged intestinal wall and that the transfer of TLR2-, TLR3-, TLR4-, TLR7-, or TLR9-deficient neutrophils into wild-type mice resulted in a less severe aGVHD.^122^ Consistent with these findings, a prospective collection of intestinal tissue biopsies from 37 patients undergoing alloHSCT, with or without aGVHD, made it clear that the severity of intestinal aGVHD strongly correlates with the number of neutrophils in GVHD lesions, as well as with anti-nitrotyrosine staining to detect oxidative damage.^122^ Overall, these data indicate that total body irradiation (TBI) and chemotherapy delivered in the context of conditioning regimes lead to translocation of bacteria into intestinal tissues, with a consequent recruitment of neutrophils. Local activation of neutrophils by TLR ligands and neutrophil-mediated tissue damage by ROS further promote T-cell differentiation into effector T cells, causing aGVHD. In a more recent in vivo study, Hulsdunker et al.^123^ have demonstrated that mouse neutrophils may also participate in aGVHD pathogenesis by functioning as APCs. In fact, by using a photoconverter reporter system, these authors were able to demonstrate that neutrophils, previously exposed to 405 nm light in the ileum for 7 min after TBI conditioning, migrated to draining mLNs 1–2 days later.^123^ Interestingly, neutrophil migration was dependent on the presence of translocated bacteria (or their components) in mLNs, as demonstrated by the increased number of bacterial 16S RNA copies in TBI-treated mice compared with controls, as well as by the reduced number of neutrophils in the mLNs of antibiotic-treated mice.^123^ Interestingly, neutrophils were found to colocalize with donor-derived T cells in mLNs and therein to present antigens on major histocompatibility complex-II (MHC-II).^123^ Accordingly, neutrophil depletion by anti-Ly6 antibodies was associated with reduced T-cell proliferation in mLNs.^123^ Notably, ruxolitinib (the JAK1–JAK2 inhibitor), an effective drug against GVHD in clinical settings, was shown to interfere with both neutrophil migration to mLNs and antigen presentation.^123^ Moreover, ruxolitinib-treated mice were found to display a lower absolute number of neutrophils in mLNs, with lower MHC-II expression, than vehicle-treated mice.^123^ Very recently, the same group^124^ has demonstrated reduced aGVHD-related mortality in mice undergoing active or passive immunization against poly-N-acetylglucosamine (PNAG), a conserved microbial surface polysaccharide that is expressed by various pathogens in murine aGVHD models. Interestingly, the targeting of PNAG reduced uncontrolled neutrophil activation in mouse ileum, in turn favoring the elimination of opsonized bacteria.^124^ Finally, in mouse models of aGVHD, allogeneic T-cell-derived GM-CSF was found to license donor-derived phagocytes, including neutrophils, to damage host tissues via the release of inflammatory mediators and ROS.^125^

Taken together, the studies reported above^122,123^ confirm the role of conditioning regimen-induced tissue injury as an early trigger of aGVHD, resulting in the release of DAMPs and MAMPs. In this scenario, neutrophils seem to react very efficiently to tissue damage and bacterial translocation by amplifying damage^122^ and promoting allogeneic T-cell activation.^123^ Despite this convincing experimental evidence, it remains to be established whether reducing the intensity of some conditioning regimens may limit the extent of neutrophil activation in humans. Furthermore, it would be important to clarify how long patient and graft neutrophils can survive after conditioning regimens (albeit in an inflammatory environment caused by the release of proinflammatory cytokines^126^). It would also be important to clarify whether residual patient and reconstituted donor-derived neutrophils are able to sustain potent inflammatory activities despite the administration of immunosuppressive prophylactic agents.

### Chronic GVHD (cGVHD)

cGVHD is a major cause of late nonrelapse morbidity and mortality in alloHSCT patients.^127^ Clinically, cGVHD is a pleiotropic, multiorgan syndrome involving tissue inflammation and fibrosis, often resulting in permanent organ dysfunction.^127^ The pathophysiology of cGVHD involves multiple, distinct interactions among alloreactive and dysregulated T and B cells and innate immune populations, including macrophages, DCs and neutrophils, that culminate in the initiation and propagation of pro-fibrotic pathways.^128^ Ocular cGVHD is relatively frequent in alloHSCT patients.^129^ Symptoms and signs of ocular cGVHD include ocular discomfort, tear deficiency with corneal and conjunctival epitheliopathy, eyelid disease with Meibomian gland (MG) atrophy, ocular surface inflammation and cicatrization, and superior limbic keratoconjunctivitis.^129^ Neutrophils are hypothesized to be involved in ocular cGVHD pathogenesis mainly via their release of NETs. In fact, in a study including 30 patients with ocular cGVHD, 18 without and 20 HDs, An et al.^129^ showed by immunofluorescence staining that NETs were abundant in mucocellular aggregates and ocular surface washings of patients affected by ocular cGVHD. Accordingly, MPO- and NE-DNA complexes and NET-associated proteins [NE, MPO, CXCL8, TNF-α, brain-derived neurotrophic factor (BDNF), oncostatin M (OSM), neutrophil gelatinase-associated lipocalin (NGAL), and LIGHT/TNFSF14] were detected at higher levels in ocular surface washings from patients with ocular cGVHD than in those from patients without ocular cGVHD and from HDs.^129^ Notably, the same proteins and cytokines (with the exception of TNF-α) were detected in supernatants from PMA-stimulated human neutrophils undergoing NETosis.^129^ In addition, ocular cGVHD patients who had an excess of neutrophils over epithelial cells in ocular surface washings had greater disease severity.^129^ In cultured human corneal epithelial cells, the addition of PMA-stimulated neutrophils as a source of NETs delayed the closure of scratch wounds and induced epithelial mesenchymal transition.^129^ In vitro, NETs also increased the proliferation of cultured human conjunctival fibroblasts, induced a robust myofibroblast transformation, caused the contraction of collagen matrices, and significantly reduced MG cell proliferation and differentiation.^129^ Importantly, in vivo experiments showed that prolonged application of NETs to murine corneas caused epitheliopathy and delayed epithelial wound healing.^129^ In addition, disruption of NETs with heparin diminished all NET-mediated effects in vivo and in vitro.^129^ Notably, the same authors were able to identify the NET-associated enzymes or cytokines most responsible for NET-mediated pathogenic effects, indicating OSM and LIGHT/TNFSF14 as the main inducers of epitheliopathy and T-cell proliferation and NGAL as the main inhibitor of MG epithelial cell proliferation and differentiation.^129^

As previously stated, neutrophils may exert opposite functions depending on the stimuli they receive. In keeping with this notion, recent evidence has shown that neutrophils of cGVHD patients acquire immunosuppressive properties following extracorporeal photopheresis (ECP), an immunomodulatory procedure used for the treatment of steroid-resistant aGVHD or cGVHD.^130^ ECP consists of the apheresis of leukocytes from patient whole blood and their subsequent reinfusion after chemoirradiation with the photosensitizing agent 8-methoxypsoralene (8-MOP) and UVA light.^130^ Although the therapeutic mechanisms of ECP are only partially known, T cells and DCs have been traditionally regarded as the main targets.^130^ However, Franklin et al.^131^ have recently demonstrated that neutrophils, accounting for the majority of leukocytes treated during ECP, undergo some level of apoptosis and lose their inflammatory properties upon chemoirradiation. Accordingly, neutrophils obtained from chemoirradiated buffy coats of cGVHD patients displayed an increased apoptotic rate after culture for 24 h, while their residual viable fraction showed a loss of activation markers (CD16, CD54 and, to a lesser extent, CD11b).^131^ Upon 24 h of in vitro stimulation with LPS, neutrophils from chemoirradiated buffy coats of treated patients were found to reduce the secretion of CXCL8 and CCL4 and increase the release of Arg-1 compared to nonchemoirradiated neutrophils from HDs.^131^ Consistently, compared with neutrophils isolated from cGVHD patient peripheral blood prior to ECP, neutrophils isolated 24 h after ECP showed a higher rate of apoptosis and also lower secretion of CCL4 and increased release of Arg-1 after 24 h in vitro stimulation with LPS^131^. Importantly, the neutrophils isolated 24 h after ECP were able to suppress in vitro the proliferation of both autologous and heterologous T cells.^131^ In agreement with ex vivo findings, in vitro treatment of HD neutrophils with 8-MOP and UVA accelerated their spontaneous apoptosis and reduced the expression of activation markers (i.e., CD16 and CD54) by the residual viable neutrophils, therefore reproducing ex vivo data.^131^ Moreover, following a 24-h in vitro stimulation with PMA, chemoirradiated neutrophils from HDs displayed a reduced ROS and NO production capacity compared to controls, while a 24-h culture with LPS increased their release of Arg-1.^131^

Interestingly, subsequent in vitro studies from the same authors^132^ demonstrated that 8-MOP- and UVA-treated neutrophils are unable to activate autologous DCs, failing to induce their upregulation of CD80, CD83, and programmed-death ligand 1 (PD-L1) and release of IL-10, TNF-α and IL-12p70. In the same experimental setting, neutrophils were also found to impair the LPS-induced and IFNγ-induced activation of DCs.^132^ Consistently, DCs previously cultured with chemoirradiated neutrophils showed a reduced allostimulatory activity in mixed lymphocyte reaction.^132^ Similar results were observed when culturing chemoirradiated neutrophils with other APCs (i.e., monocytes and macrophages).^132^ Overall, the two studies described above^131,132^ indicate that neutrophils of patients undergoing ECP acquire the capacity to control cGVHD by inhibiting T cells either directly or indirectly by hampering the activation of APCs.

Rieber et al.^43^ had previously described in the peripheral blood of patients affected by aGVHD or cGVHD a population of functionally active CD33^high^CD66b^high^HLA-DR^low^IL-4R^+^CXCR4^+^ LDNs/PMN-MDSCs that significantly expanded after ECP treatment. Nonetheless, based on the great prevalence of NDNs over LDNs in the apheretic leukocytes of patients undergoing ECP,^131^ it can be speculated that NDNs, rather than LDNs/PMN-MDSCs, represent the main effectors of the myeloid-mediated immunosuppressive activity of ECP.

### Cytomegalovirus (CMV) reactivation

Neutrophils are involved in antiviral immune responses, but the effects they mediate may be either helpful or detrimental to the host, depending on the type of viral infection.^133^ Human CMV (HCMV) is a β-herpesvirus that establishes a lifelong latent infection in BM-resident CD34^+^ cells and CD33^+^ myeloid progenitors in infected immunocompetent individuals.^134^ HCMV reactivation represents the most frequent opportunistic infection after alloHSCT^134^ and a predictor of poor outcomes.^135,136^ In fact, recipients of alloHSCT with a positive serology for HCMV are at increased risk for HCMV reactivation and for early and late nonrelapse mortality.^135,136^ Interestingly, the data in the literature indicate that, although not effective in sustaining a full lytic viral replication,^137,138^ HCMV has the capability to exploit and evade the host immune system in order to ensure its own dissemination and long-term survival.^139,140^ In this perspective, neutrophils have been shown to participate in HCMV dissemination as carriers, following their recruitment to HCMV-infected sites.^141^ As such, in the transendothelial migration assay, human neutrophils were found to migrate preferentially toward supernatants from HCMV-infected endothelial cells compared with controls, further expressing the CMV structural protein pp65 upon coculture (i.e., cell-to-cell contact) with HCMV-infected endothelial cells.^141^ Although CXCL8 released by HCMV-infected endothelial cells was initially indicated as the main neutrophil chemoattractant, subsequent sequencing studies on HCMV (Toledo strain) identified two viral genes, namely, *UL146* and *UL147*, with sequence motifs reminiscent of human CXCL8.^142^ In in vitro studies, the product of *UL146*, vCXCL-1, acted as a potent neutrophil chemoattractant^142^ that was also found to target neutrophils through CXCR1 and CXCR2 binding.^143^ More recently, an in vivo study conducted in a mouse model infected with recombinant mouse CMV (MCMV) expressing HCMV-encoded vCXCL-1 demonstrated that vCXCL-1 is able to increase MCMV dissemination, thus representing a virulence factor.^140^ In addition to their role as carriers, human neutrophils have been shown to display a remarkable increase in survival in response to HCMV.^144^ Accordingly, upon in vitro exposure to the clinical HCMV isolate Merlin, neutrophils were found to delay their own apoptosis through the activation of NF-kB and ERK1/2 and the consequent stabilization of the anti-apoptotic protein MCL-1.^144^ In addition, virus-free supernatants from human neutrophils (but not from monocytes) infected with the same HCMV strain were found to exert a stronger anti-apoptotic effect toward freshly isolated neutrophils in vitro compared to controls, likely due to a higher concentration of pro-survival molecules such as TNF-α, IL-6, and CXCL8.^144^ In vitro experiments have also shown that human neutrophils do not kill latently HCMV-infected monocytes owing to a suppressed secretion of neutrophil chemoattractants (i.e., S100A8/A9) by infected monocytes themeselves.^145^ However, in contrast to the supportive role of neutrophils toward CMV, an in vivo study conducted in a mouse model of primary MCMV infection reported instead that neutrophils exert TNF-related apoptosis-inducing ligand (TRAIL)-mediated anti-MCMV activity in infected tissues upon their CXCL-1-mediated recruitment.^146^

Despite the accumulating findings regarding potential interactions between neutrophils and CMV, both in human and in mouse models, much remains to be defined and clarified. In particular, to the best of our knowledge, no ex vivo studies analyzing ultrapure neutrophils from alloHSCT patients, with or without HCMV reactivation, are currently available. These studies would help to unequivocally define, for instance, the pattern of cytokines and chemokines expressed and released by neutrophils carrying HCMV, their phenotypic features or unknown functional responses. Similarly, no studies have analyzed in detail whether the clinically known relationship between HCMV reactivation and GVHD^147^ may be explained by an increased survival^144^ and proinflammatory attitude^142^ of HCMV-infected neutrophils. In our opinion, these latter studies are urgent, as neutrophils represent the first reconstituting cells and are almost the only players in the immune system in the first months after alloHSCT, when HCMV usually reactivates and aGVHD occurs.

## Conclusions and perspectives

**Fig. 6:**
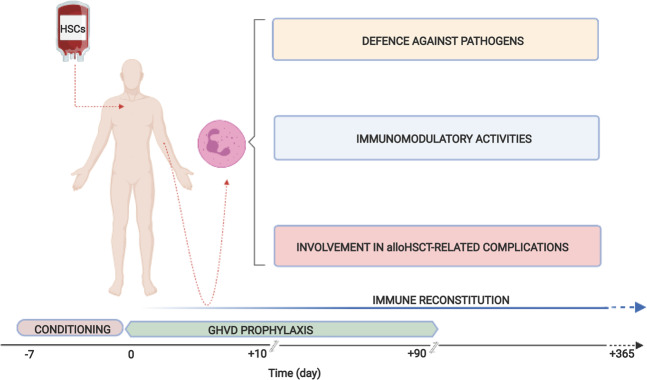
Functions of neutrophils described following alloHSCT. The figure lists all neutrophil functions observed during the immune reconstitution period, reviewed in the text according to published literature

It is evident that neutrophils participate throughout the entire alloHSCT procedure, as well as in alloHSCT-related complications (Fig. 6). In fact, neutrophils are present in graft products, mostly  in G-CSF-mobilized PBSCs, and rapidly reconstitute after HSC infusion as almost fully competent cells. However, despite data herein recapitulated, much remains to be explored with regard to neutrophil functions in the alloHSCT setting. In fact, the role played by neutrophils in human alloHSCT has been largely overlooked, especially in light of the multiple, novel biological activities that have been uncovered in the last years^11^ (Fig. 1). As already mentioned in this review, studies are awaited to establish the effective proportions and the immunomodulating potentialities of immature and mature neutrophils copurified in G-CSF-mobilized HSC products, their precise survival, their possible response and/or contribution to the cytokine storm, and the type and amounts of DAMPs/MAMPs they encounter upon infusion in the recipient, depending on conditioning regimen intensity. Immune reconstitution is characterized by the rapid restoration of a normal count of neutrophils with a mature phenotype. However, the functions of these cells should probably also be evaluated in terms of the types and plasma levels of the immunosuppressive drugs used for GVHD prophylaxis and/or treatment, of the eventual G-CSF administration and of viral infections. In addition, attention should be paid to the interactions that neutrophils may establish with the different types of reconstituting cells, whose compositions (i.e., from innate immune cells to progressively more competent adaptive immune cells) may vary over time. Neutrophils may also interact with cells infused after alloHSCT for therapeutic purposes, such as third-party MSCs and donor lymphocytes, which are used in the treatment of GVHD and primary disease relapse, respectively. Finally, the contributions of neutrophils to many alloHSCT-associated complications largely remain to be explored.

In conclusion, it is evident that neutrophils may contribute to alloHSCT outcomes in multiple ways, which are likely to vary depending on the characteristics of each patient. Based on the studies available, it seems reasonable to say that, under alloHSCT settings, neutrophils are much more than simple phagocytic cells. Therefore, future studies are awaited to elaborate the still untold story of neutrophils in alloHSCT and the eventual possibility of modulating their functions for therapeutic purposes.
